# The impact of contact tracing and testing on controlling COVID-19 outbreak without lockdown in Hong Kong: An observational study

**DOI:** 10.1016/j.lanwpc.2021.100374

**Published:** 2022-01-15

**Authors:** Hsiang-Yu Yuan, Colin Blakemore

**Affiliations:** a Department of Biomedical Sciences, Jockey Club College of Veterinary Medicine and Life Sciences, City University of Hong Kong, Hong Kong SAR, China; b Centre for Applied One Health Research and Policy Advice, Jockey Club College of Veterinary Medicine and Life Sciences, City University of Hong Kong, Hong Kong SAR, China; c Department of Neuroscience, City University of Hong Kong, Hong Kong SAR, China; d Hong Kong Institute for Advanced Study, City University of Hong Kong, Hong Kong SAR, China

## Abstract

**Background:**

Maintaining effective contact tracing to control COVID-19 is challenging. Rapid growth in the number of infected cases can overload tracing and testing capacity, resulting in failure to trace contacts and delays in confirming an infection until after symptom onset (confirmation delay), hence increasing transmissibility. A substantial outbreak in Hong Kong, which was suppressed with non-pharmaceutical interventions (NPIs), provided an opportunity to assess the impact of overloading contact tracing and of efforts to improve its efficiency.

**Methods:**

Using epidemiological-link (epi-link) data, we calculated the probability and duration of confirmation delay for cases with and without an epi-link, among all 3,148 confirmed cases between 5 July and 15 August 2020. Logistic regression was performed to determine the relationship between the number of recently confirmed infections and the probability of confirmation delay for epi-linked (contact-traced) cases. We estimated the impact on this relationship of targeted testing of at-risk groups.

**Findings:**

The probability and duration of confirmation delay were associated with the rise in daily case number during growth of the outbreak. The proportion with confirmation delay among contact-traced cases increased from about 60% to nearly 85% as the number of cases grew from 1 to 50 per day (p-value = 0.003). The subsequent introduction of testing services for at-risk groups substantially reduced the proportion and it did not approach 85% again until the daily number of cases exceeded 125. This 2.5-fold improvement in capacity contributed crucially to suppression of the outbreak.

**Interpretation:**

The number of recently confirmed infections is an indicator of the load on the contact-tracing system, the consequence of which can be assessed by the probability of confirmation delay. Measures to monitor and improve contact-tracing efficiency, alongside social distancing interventions, can enable outbreaks to be controlled without lockdown.

**Funding:**

City University of Hong Kong and Health and Medical Research Fund.

**Research in context**

**Evidence before this study**

We searched PubMed, bioRxiv, and medRxiv for articles published from 1 January, 2020 to 31 March, 2021, with the following keywords: (“2019-nCoV” OR “COVID-19” OR “SARS-CoV-2”) AND (“contact-tracing efficiency” or ”effectiveness of contact tracing” or ”confirmation delay” or “isolation delay”). 7 recent population-level modelling or simulation studies of COVID-19 have demonstrated theoretically the value of minimizing confirmation delay. However, no previous observational study has documented a change in the probability and duration of confirmation delay associated with an increase in number of cases, or determined to what extent different approaches of tracing and testing can mitigate such effects.

**Added value of this study**

We present the dynamics of the proportion of COVID-19 cases that were traced and of delays in confirmation during a substantial outbreak that was suppressed by NPIs without lockdown. We found that: i) restoring social distancing measures without maintaining tracing and testing efficiency was not enough to prevent growth of the outbreak; ii) a rise in number of daily cases increased the probability of confirmation delay among contact-traced cases; iii) testing at-risk groups reduced the probability and the duration of confirmation delay among contact-traced cases.

**Implications of all the available evidence**

Our results demonstrate the importance of efficient contact tracing and testing for the control of outbreaks. Monitoring the proportion of cases with an epi-link, the fraction with confirmation delay and the distribution of delay provides a measure of efficiency. Targeted testing of at-risk groups can be highly effective in improving efficiency. An agile and efficient contact-tracing system, combined with selective strengthening of social distancing measures, has been shown to be capable of suppressing a substantial outbreak without radical lockdown.

Alt-text: Unlabelled box

## Introduction

Although there is now real hope that vaccination will eventually terminate the COVID-19 pandemic, new outbreaks are likely to occur, perhaps for many years. Knowing how best to control outbreaks with non-pharmaceutical interventions (NPIs) is critically important, in order to avoid damaging lockdowns.

Alongside social distancing measures, maintaining or increasing the efficiency of contact tracing and testing plays a crucial role in constraining the spread of infection.^1^, ^2^, ^3^ However, rapid growth in the number of infected cases during expansion of an outbreak can overwhelm contact-tracing capacity,^4^ which might reduce efficiency. Prolonging the time taken for tracing and testing will increase the probability of confirmation being delayed until after symptom onset (**confirmation delay**). If the cases are isolated after confirmation, this delay is also called onset-to-isolation delay.^1^ Longer delay leads to higher transmissibility,^1^, ^2^, ^5^ so it is important to identify interventions that can reduce confirmation delay. Change in load on the contact-tracing system, which depends on the number of recently confirmed cases, must therefore be considered. This demands a better understanding of the relationship between the dynamics of outbreaks and confirmation delay.^6^, ^7^

The so called ‘third wave’ in Hong Kong is one of the few examples of a significant national outbreak of COVID-19 that has been brought under control without a full lockdown. Observational data from this outbreak provide an opportunity to ask whether NPIs contributed significantly to moderating confirmation delay.

The trigger for the third wave has been attributed to imported cases that were exempted from quarantine (such as aircrew and sailors) mainly from Asian countries,^8^, ^9^, ^10^ the impact of which was exacerbated by substantial relaxations of social distancing measures. After 21 consecutive days without a single local case, the primary/index case for the third wave occurred on 5 July 2020.

During the outbreak, efforts were made to trace and test the close contacts of infected patients. When contact-traced cases (including backward-traced) were confirmed, they were isolated in hospital and reported with the label ‘epi-link’ (epidemiological link), indicating that their contact sources had been successfully identified. Even though social distancing measures were strengthened, an alarming surge led to more than 2,000 local cases within a month (in a population of about 7.5 million), many of which could not be linked to an infection source.

Faced with a growing number of cases that could not be traced, the Hong Kong government initiated a ‘targeted group testing scheme’ for certain high-risk individuals.^11^ Within a few weeks, the outbreak was successfully controlled. It is important to know whether targeted testing restored contact-tracing efficiency, reduced confirmation delay and hence helped to suppress the outbreak.

The present study of observational data from the third wave was aimed at exploring the relationship between the dynamics of transmission and confirmation delay, in relation to the NPIs that were deployed. These results have informed our comprehensive modelling of the outbreak and quantitative assessment of the impact of each NPI.^12^

## Materials & methods

### Data sources

We retrieved the dates of symptom onset and confirmation for each newly imported and local case of COVID-19 between 17 June and 15 August 2020 from the Hong Kong Centre for Health Protection.^13^ Transmission clusters were classified as restaurants, housing estates, shopping areas, and workplaces. We collected the daily number of total passengers from the Hong Kong Immigration Department^14^ during the study period. The number of visitors who were exempted from compulsory quarantine was acquired from records of medical surveillance orders for quarantine-waived travellers issued by the Department of Health.

### Estimating effects of targeted group testing

A logistic regression model, with a quasi-binomial distribution to deal with overdispersion, was used to estimate the effects of targeted group testing on the delay in confirmation. We calculated the probability of having such delay among contact-traced cases, given the number of recently confirmed cases:

(1) $$Y_{t}∼Quasi\;Binomial\left(\mu_{t}\right)$$

(2) $$log\left(\frac{\mu_{t}}{1-\mu_{t}}\right)=\alpha +\beta \times Cases_{t}+\gamma \times \left(Cases_{t}\cdot D_{t}\right)$$

where $Y_{t}$ is the observed proportion of confirmation delay among contact-traced cases at day t, distributed with the expected probability of confirmation delay $\mu_{t}$. Quasi-binomial model was used to model the overdispersed Binomial data. $log(\frac{\mu_{t}}{1-\mu_{t}})$ is the logit of the probability. α is the intercept and β is the regression coefficient. $Cases_{t}$ is the average daily number of cases reported in the current 7-day period, centered at day t (chosen to refer to the time interval of one generation in an outbreak^15^). $D_{t}$ is a dummy variable used to account for the status without (D=0) or with an intervention (D=1). γ is the regression coefficient that changes the slope of the relationship between Cases and the response when the intervention is used. The baseline probability of confirmation delay (when the average daily number of case is approaching zero) is assumed to be the same without and with the intervention.

### Determining capacity before and after an intervention

The maximum average daily number of cases (MaxCases) that can be tolerated before the probability of confirmation delay exceeds a particular value indicates the capacity of tracing and testing for that threshold. After the intervention is implemented, the maximum daily number that can be tolerated before the same threshold is exceeded can be derived by letting $\beta \times MaxCases=\left(\beta +\gamma \right)MaxCases^{I}$, where the superscript I indicates the period after the intervention has been introduced. If the intervention reduces the odds of confirmation delay, *γ* will be negative. The maximum number is increased by the ratio:

(3) $$\frac{MaxCases^{I}}{MaxCases}=\frac{\beta }{\left(\beta +\gamma \right)}$$

when γ is negative. A larger ratio indicates that the system is more robust in maintaining efficiency against changes in load.

### Role of the funding source

The sponsor of the study had no role in study design, data collection, data analysis, data interpretation, or writing of the report. The corresponding author had full access to all the data in the study and had final responsibility for the decision to submit for publication.

## Results

After a period without local infections, social distancing measures were relaxed, as follows:

• **Relaxation 1 (R1)**, from 19 June 2020. The maximum number permitted to gather in public places was increased from 8 to 50.^16^
• **Relaxation 2 (R2)**, from 2 July 2020. The maximum in places of entertainment was raised from 50% to 80% of capacity.^17^

**Figure 1:**
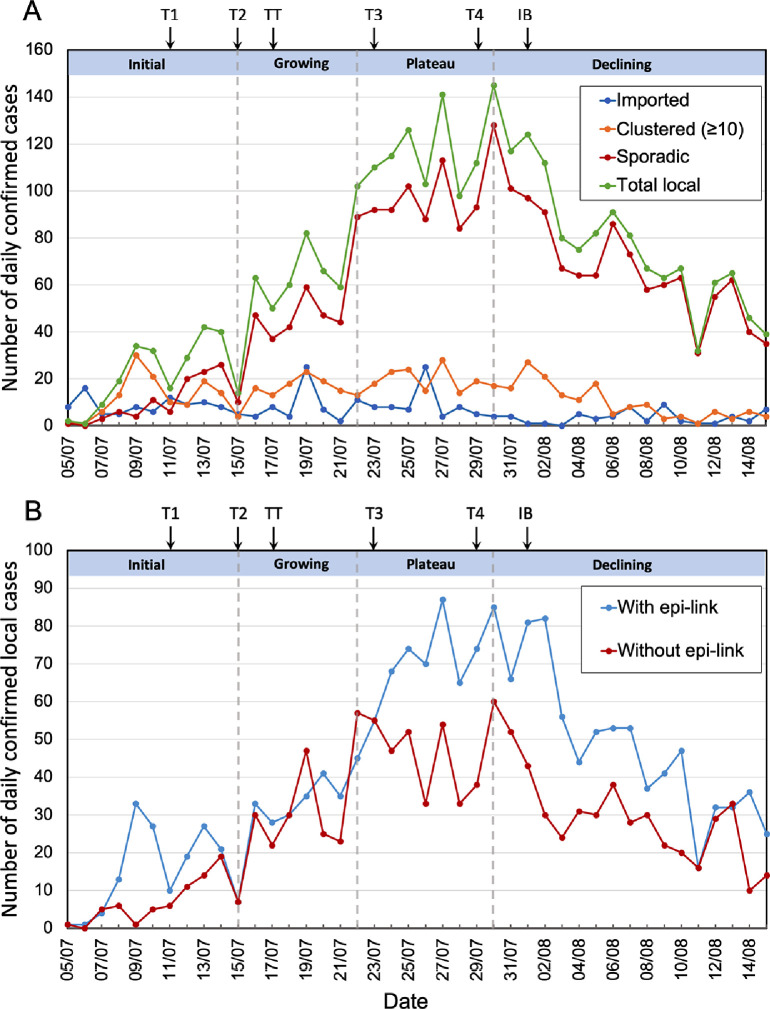
(A) Number of confirmed cases per day during the third wave, starting with the first reported case on 5 July. Numbers are plotted separately for Imported cases (blue); Clustered local cases, defined as cases from clusters of 10 or more linked to the same infection source (orange); Sporadic local cases, without an epi-link or from clusters of fewer than 10 cases (dark red); and Total local cases (green). Four epidemic phases are defined: **Initial** (5 July - 15 July), **Growing** (15 July - 22 July), **Plateau** (22 July - 30 July) and **Declining** (30 July - 15 August). (B) Daily confirmed local cases plotted separately for individuals with an epi-link to an earlier case (blue) and those without an epi-link (dark red). Arrows above the graphs indicate the dates of introduction of NPIs (for abbreviations see main text). Targeted group testing (TT) is shown at 17 July, because that is the launch date for the major component of this scheme (see text).

The primary/index case was reported on 5 July 2020. Daily numbers of local cases rose gradually until 15 July, and then more rapidly to 30 July. After a decrease in the rate of growth, from 22 to 30 July, numbers started to fall. The third wave can be considered in four epidemic phases: **initial** (5 July - 15 July), **growing** (15 July - 22 July), **plateau** (22 July - 30 July) and **declining** (30 July - 16 August) (Figure 1A).

**Figure 2:**
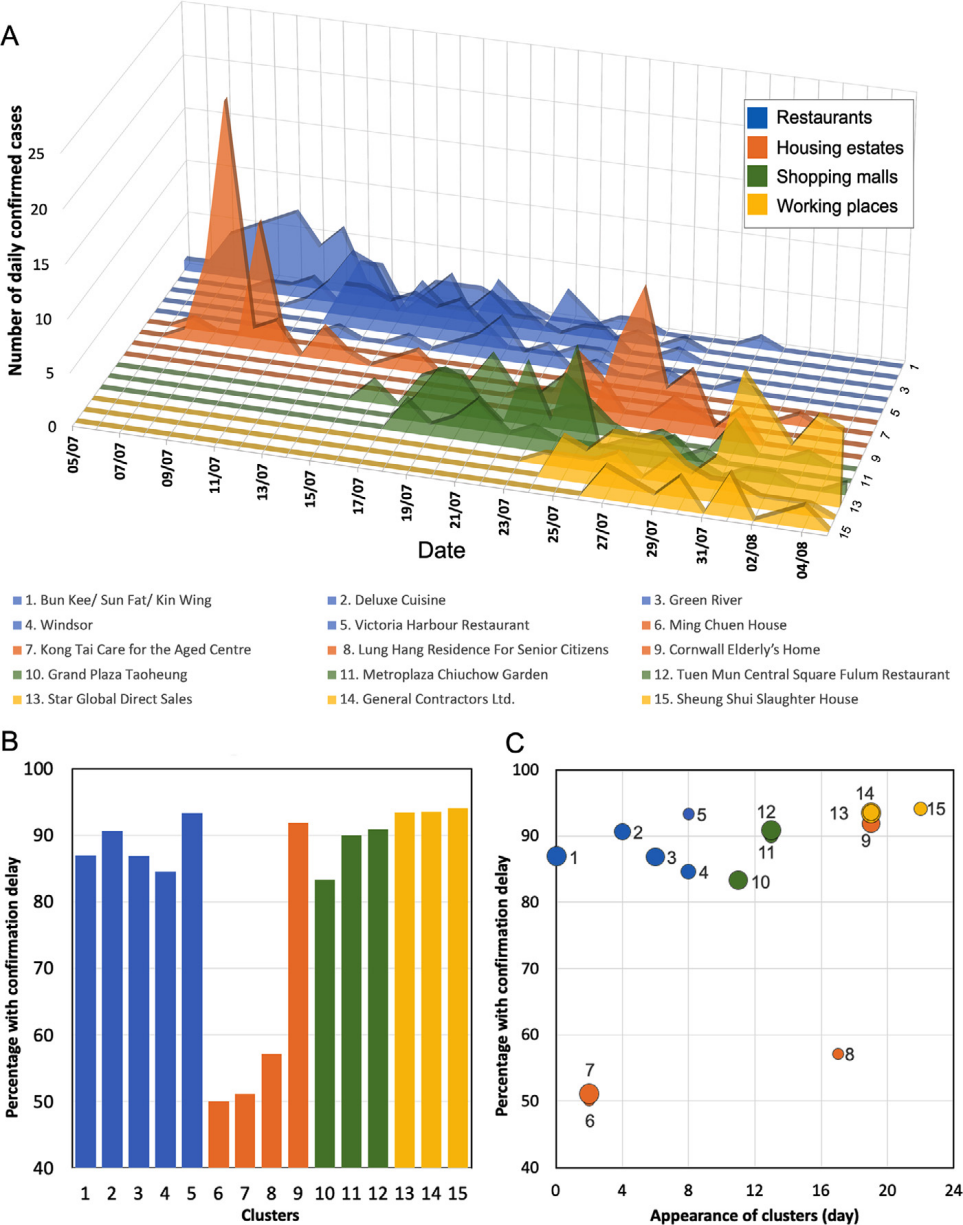
Evolution of transmission clusters and occurrence of confirmation delay (i.e. infection confirmed after symptom onset). (A) Daily number of cases of each of the 15 large transmission clusters that emerged before the peak of the outbreak. The different classes of clusters are shown in different colours (see legend) (B) Percentage of total number of cases with confirmation delay in each transmission cluster. (C) The temporal trend in confirmation delay among these clusters. The ordinate shows the percentage of cases with confirmation delay in each cluster. Each point plots the time of appearance of a cluster, in days after the start of the outbreak. The area of each point is proportional to the total number of cases in the cluster.

The outbreak was likely seeded by undetected imported cases,^8^, ^9^, ^10^ estimated to have been about one per day, among visitors exempted from quarantine (see Figure S1 and Supplementary Material), and then ignited by transmission clusters (Figure 2). During the initial phase, cases were mainly associated with seven large clusters (defined as ten or more cases with an epi-link to the same infection source), identified by contact tracing and investigation (Figures 1A, 2 A).

Social distancing measures were tightened in four steps from 11 July until 29 July:

• **Tightening 1 (T1)**, from 11 July. The maximum number per table in catering premises was reduced to 8 and the maximum in places of entertainment was reduced from 80% to 60% of capacity.^18^
• **Tightening 2 (T2)**, from 15 July. Numbers gathering in public places and at restaurant tables were reduced to 4. The maximum in places of entertainment was reduced to 50% of capacity.^19^
• **Tightening 3 (T3)**, from 23 July. Mandatory mask-wearing was extended from public transport to all indoor public places.^20^
• **Tightening 4 (T4)**, from 29 July. The maximum gathering number was reduced from 4 to 2 and evening service in restaurants was banned.^21^

After 11 July, mobility, i.e. the number of visits and time spent in places of retail & recreation and in public transportation stations, started to decrease (Table S1, Figure S2). Despite the introduction of social distancing measures (T1 + T2) that were stricter than before relaxation (Table S1), and notwithstanding this lower mobility, the daily number of confirmed cases increased rapidly from 15 to 22 July (Figure 1A). The effect of social distancing measures may not be clearly observed immediately due to confirmation delay. During this period, numbers associated with major transmission clusters remained fairly constant and the surge predominantly involved ‘sporadic’ cases, defined as individual cases without an epi-link (i.e. not identified by contact tracing) or associated with small linked groups (≤10 per cluster).

After an inflection on 22 July, daily numbers became fairly steady for more than a week. Following established conventions,^22^ we call this phase a ’plateau’. However, numbers did continue to increase slowly, and the highest daily number (145 local cases, including 128 sporadic cases) occurred on 30 July. During this period, social distancing was further strengthened (T3 and T4). Mobility continued to decrease until the end of July (Figure S2). Thereafter, daily numbers fell from 30 July to 15 August, followed by low numbers for a few more weeks (around 20 per day). The strengthening of social distancing must have contributed to this success, but here we focus on understanding the role of contact tracing and testing.

### Inefficiency of contact tracing during the outbreak expansion

**Figure 3:**
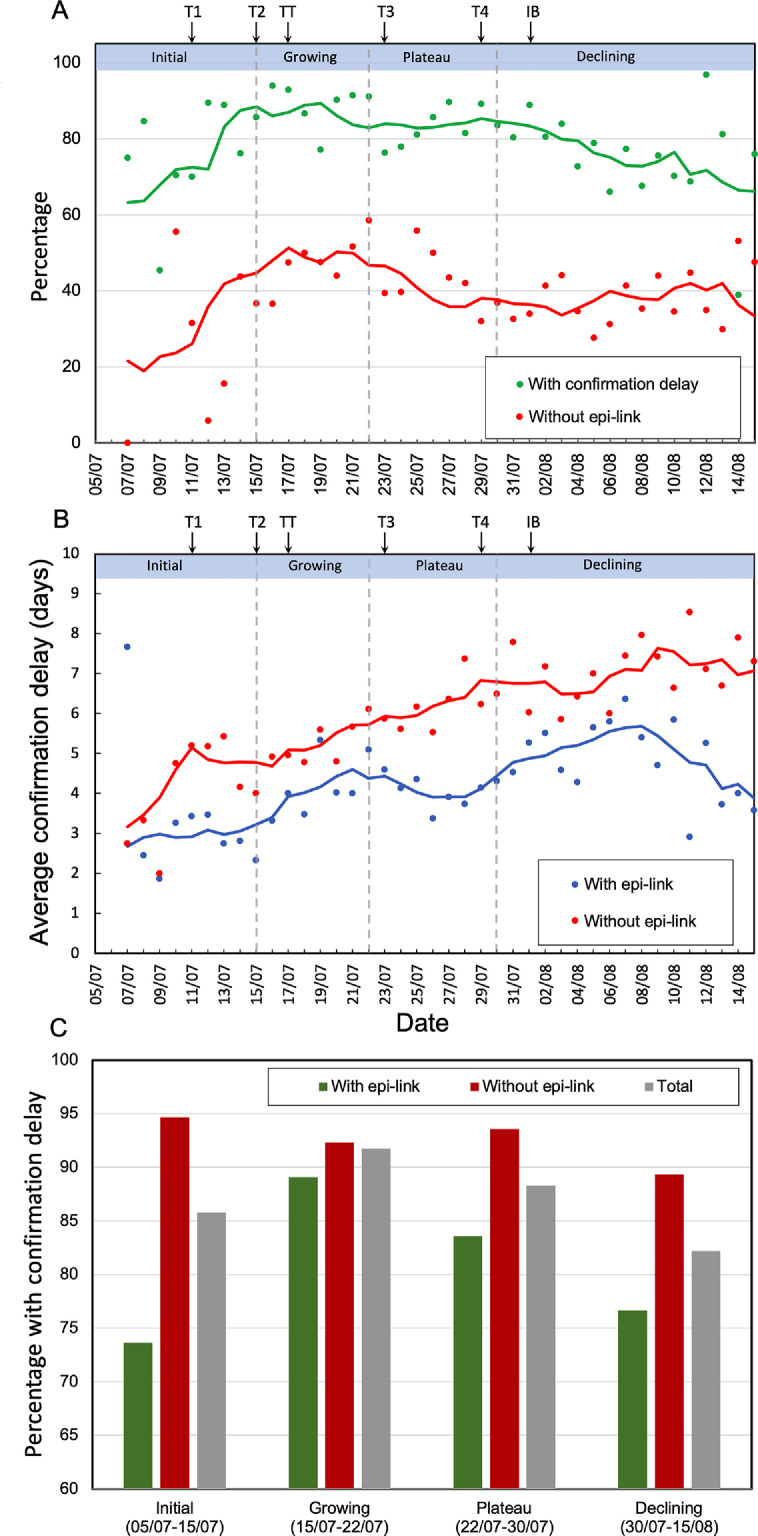
Dynamics of indicators of contact tracing inefficiency. (A) Red circles represent the proportion of all cases without an epi-link and green circles the proportion of contact-traced cases with confirmation delay. Cases before 7 July were ignored because the small numbers (fewer than 3 per day) led to large variations. NPIs are shown by arrows, as in Figure 1, using abbreviations defined in the text. (B) Blue and red circles represent the daily mean values of confirmation delay for cases with and without an epi-link, respectively. The solid curves are sliding averages (5-day window, centred on day 3). (C) Percentage of COVID-19 cases with confirmation delay during the four epidemic phases. Green bars represent cases with an epi-link; red bars, cases without an epi-link; and gray bars, total cases.

The capacity of any contact-tracing system is likely to be challenged by a sudden surge in infected cases. The proportion of cases that are successfully traced (with an epi-link) has been taken as an indicator of contact-tracing efficiency.^1^, ^23^, ^24^ Late in the initial phase of the third wave and throughout the growing phase, the proportion without an epi-link (a measure of contact-tracing **inefficiency**) rose from less than 30% to about 50% (Figures 1B, 3A). For traced cases, the fraction with confirmation delay also rose, from below 80% (73% on average) during the initial phase to about 90% during the growing phase (Figure 3A,C). The average duration of confirmation delay for both epi-linked and unlinked cases also increased before the plateau (Figure 3B). These results indicate that contact-tracing efficiency became worse as case number rose rapidly.

To understand how confirmation delay changed within major transmission clusters during outbreak expansion, we examined the proportion of cases with confirmation delay in each transmission cluster that appeared before the peak of the outbreak (Figure 2). The earliest clusters were groups of people living in the same housing estate (or care-home), or eating at the same restaurant. A few weeks later, new clusters appeared in shopping malls and in workplaces. The proportion with confirmation delay was generally lower in the early clusters in housing estates (Figure 2B), presumably because the longer time spent in estates and the less diversified contact pattern allowed contacts to be identified and tested more quickly. However, the proportion with confirmation delay grew with the date of appearance of the housing-estate clusters and was especially high in Cluster 9, which emerged late in the growing phase (Figure 2B).

Overall, more of the later clusters showed relatively severe delay, with >90% of cases having confirmation delay (Figure 2C). A mixed-effects model of Percentagewithdelay=α+β×Appearancetime, taking account of variability between different types of clusters (i.e. each cluster considered as a group), revealed an increasing trend of percentage with confirmation delay by appearance time (regression coefficient = 1.32, p-value = 0.006 using a likelihood ratio test). Again, these results suggest that the increasing case number overloaded the capacity of the tracing and testing system.

### Improvement of tracing efficiency and reduction of delay

The proportion of all cases without an epi-link started to decrease during the plateau phase (Figure 3A). For those with an epi-link, the proportion with confirmation delay fell, from 89% during the growing phase to 83% during the plateau, and to 77%, close to the initial value, in the declining phase (Figure 3A,C). These trends suggest that the capacity for contact tracing was initially insufficient to deal with the growing case number, but that efficiency recovered during the plateau, despite the continuing increase in numbers.

Two interventions were specifically aimed at improving the efficiency of contact tracing and testing:

• **Targeted Testing (TT)**, was offered in a series of phases, each aimed at a specific at-risk group, regardless of whether they had symptoms ^11^. This testing aimed to early identify cases and was implemented along with conventional tracing and testing (Figures S3, S4). The first, for care-home staff, starting on 14 July, yielded only one positive case. The main effort began on 17 July with testing of nearly 150,000 taxi drivers and restaurant staff, followed by property management staff, transport workers, market staff, residents of estates in which cases had been discovered, etc. In those phases launched during our study period (before 15 August), 76 cases were discovered from 414,085 tests.^25^
• **Isolation capacity boosting (IB)**. A community treatment facility was opened at the AsiaWorld-Expo site on 1 August, when the outbreak was already declining.^26^

To verify whether the reduction in probability of confirmation delay during the plateau and declining phases (Figure 3A) was related to improvement in contact tracing, we compared the dynamics of delay during the four phases of the outbreak. For cases without an epi-link (mainly individuals who sought diagnosis or testing after symptom onset), the proportion with confirmation delay was very high (near 90%) and remained relatively constant (Figure 3C). For epi-linked cases, the proportion with confirmation delay varied substantially: it increased during the initial and growing phases and declined thereafter (Figure 3A,C). Segregated by whether they formed clusters, the proportions with confirmation delay reduced 18.9% in epi-linked cases belonging to clusters and 6.1% in epi-linked cases not belonging to clusters after targeted testing (TT) was introduced (Figure S5). The proportions increased again after 22 July for clustered cases, but maintained relatively stable (at least 82%) for non-clustered cases (Figure S5). The percentages reduced again after the number of cases declined. Together, these seem likely to reflect deterioration and then restoration of the efficiency of tracing and testing.

**Figure 4:**
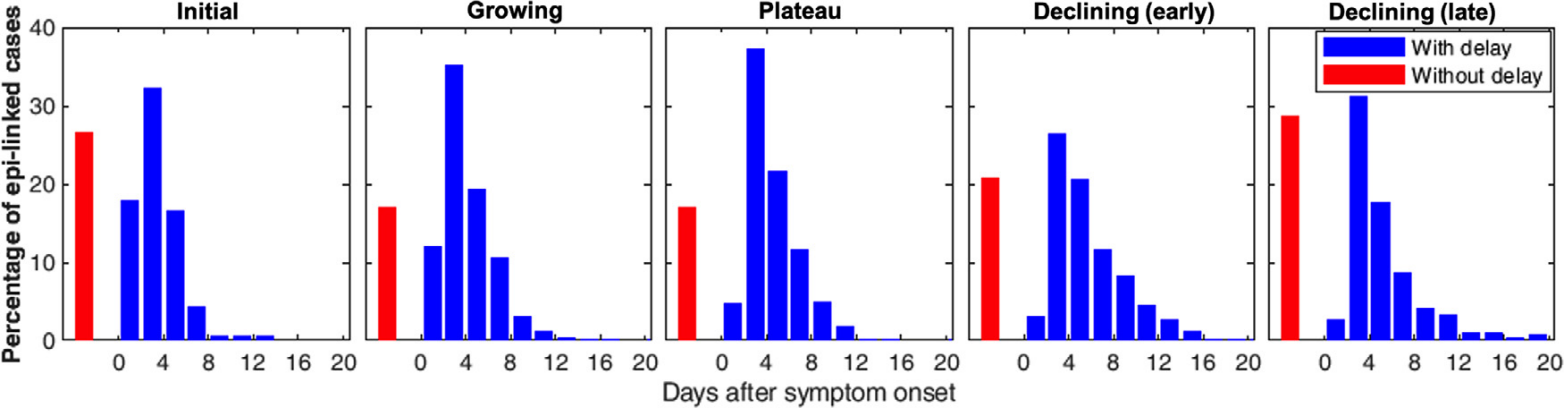
Distributions of confirmation delay during each epidemic phase. (Data for the declining phase were divided into ‘early’ and ‘late’, i.e. before and after the peak value of average confirmation delay.) Blue bars represent the percentage of epi-linked cases with confirmation delay, ranging from 0 to 20 days after symptom onset. The very small number of cases with delay >20 days are excluded from these histograms because the percentages were too small to be seen. Red bars represent the percentages of epi-linked cases without confirmation delay. Presumably, most of these cases were tested and confirmed within the 1-3 day pre-symptomatic transmission period ^33^, but some will have been true asymptomatic cases who had passed the ’normal’ incubation period without developing obvious symptoms.

Consistent with this evidence for over-loading and then recovery of contact tracing, the average duration of confirmation delay for epi-linked cases also rose during the growing phase, but decreased during the plateau (Figure 3B). However, in the early part of the declining phase (before 8 August), it rose again (Figure 3B), despite the fact that the overall percentage with delay continued to decline (Figure 3A). This apparent contradiction is explained by the distribution of confirmation delay, which became distinctly broader, indeed bimodal, early in the declining phases, with an increase in both the proportion with very long delays and the fraction without delay (Figure 4).

### Impacts of targeted group testing

The percentage of cases without an epi-link, the proportion of epi-linked cases with confirmation delay, and the duration of delay for epi-linked cases all began to fall shortly after the introduction of the targeted group testing scheme (Figure 3A,B). This leads to the hypothesis that targeted testing reduced confirmation time by improving tracing efficiency.

**Figure 5:**
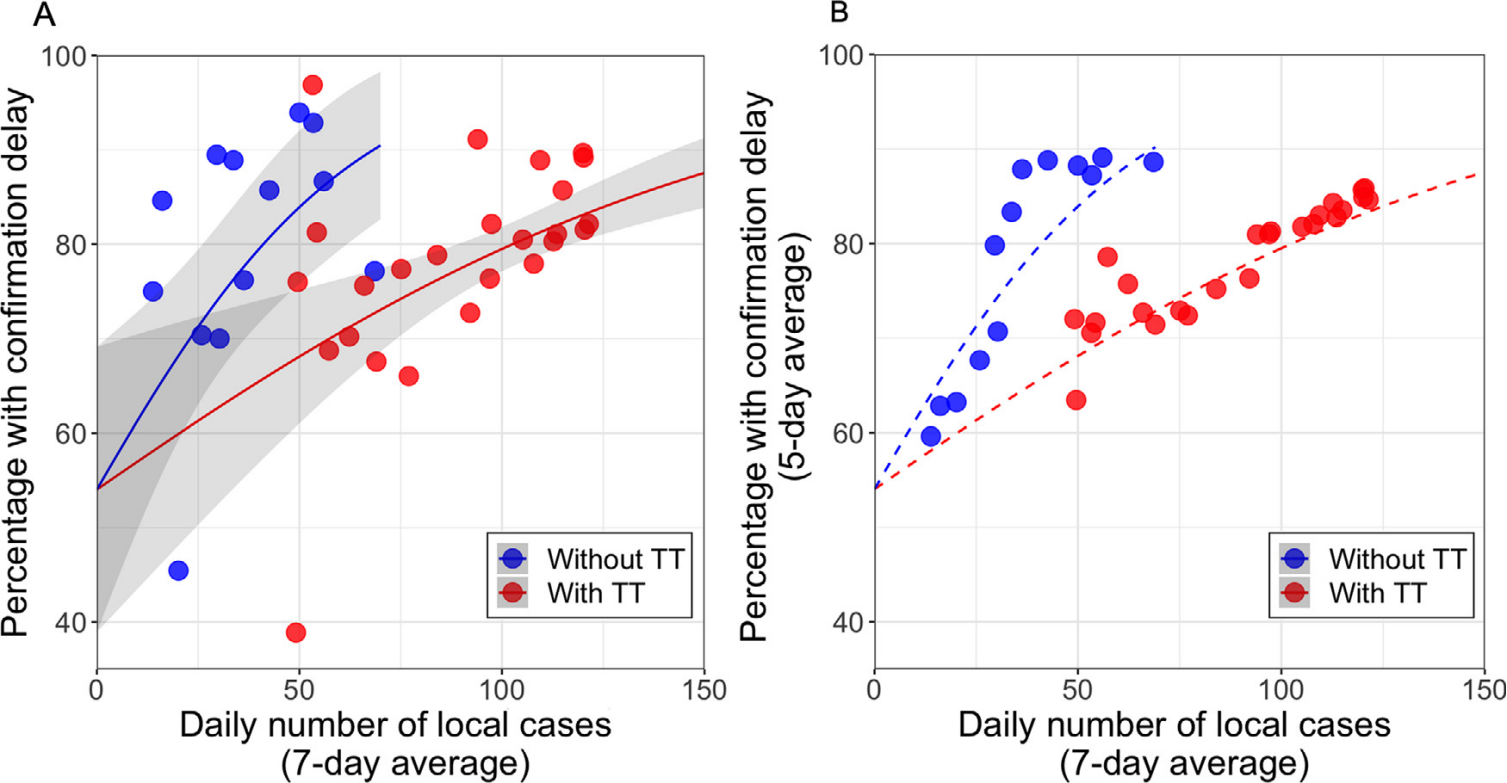
Percentage of epi-linked cases with confirmation delay as a function of the change in daily number of cases (average by week), before (blue) and after (red) the introduction of targeted group testing (TT; see Table 1). (A) Data plotted on linear coordinates. The lines are predictions from the fitted logistic regression model with standard errors for estimated coefficients in parentheses: $log\left(\frac{\mu }{1-\mu }\right)=0.162\left(0.31\right)+0.03\left(0.009\right)Cases-0.018\left(0.007\right)Cases\cdot D$ (see Methods). 95% confidence intervals are displayed. p-values of the regression coefficients of Cases and D (indication of TT) are 0.003 and 0.02. The first major phase of TT was formally introduced on a Friday (17 July) but tests were not performed until the following week. To be conservative, only data from the fifth day after introduction are included in the ’after TT’ sample. (B) To reduce the impact of daily variation in the number of reported cases, the 5-day average of percentage with confirmation delay is plotted on the ordinate, together with dashed lines showing the same fitted functions as in A. Examination of data from after the study period (between 16 and 26 August) suggests that the effect of targeted testing was maintained. Between 16 and 26 August, the daily case number was fairly constant (average 25.8; Standard Deviation (SD) = 16.5), and the mean fraction with confirmation delay was 62.2%, extremely close to the percentage for that case number predicted by the red curve above (61.5% (95% Confidence Interval(CI): 50.6 - 72.4%).

To estimate the effects of targeted group testing on confirmation delay for epi-linked cases, we used a logistic regression to compare the relationship between confirmation delay and the number of recently confirmed cases, before and after the introduction of targeted testing (see Methods and Figure 5). In both conditions, the probability of confirmation delay increased with case number from the same baseline (54%), but at a much lower rate after the introduction of targeted testing.

We also investigated whether other factors should be incorporated in our model (see Supplementary Methods and Figure S6). It is possible that more people were worried after more transmission clusters formed. However, we found that risk perception was not correlated to the delay among cases in the clusters. In comparison with the model including daily number of tests (Figure S7), the current model was the best-fitting model. Noted that social distancing measures and face mask were not included in the regression model because they are independent of confirmation delay given case numbers. The effects of these NPIs on transmissibility were investigated in a modelling study.^12^

When targeted testing was launched in mid-July, there were about 50 local cases per day (Figure 1A), with nearly 85% of epi-linked cases having confirmation delay (Figure 3A). The relationships revealed in Figure 5 suggest that targeted testing produced a dramatic decrease in the percentage with delay and, thereafter, a slower increase with case number. Indeed, Figure 5 shows that the proportion at the time of introduction (83%) was not reached again until daily case number exceed 125 – a 2.5 times increase in case number producing the same proportion. This magnitude of increase in capacity, derived by Equation 3 (see Methods), applies for the entire observed range of proportions above about 60%. The actual change in the percentage with confirmation delay in the period following TT, seen in Figure 3A, results from an interplay between the shift from the functions without to with targeted testing in Figure 5 and the rapidly rising total case number seen in Figure 1A.

A similar trend but with larger variance is seen in the relationship between the proportion of cases without an epi-link and daily case number, before and after the introduction of TT (Figure S8).

In summary, during the growing phase, the proportion with confirmation delay increased because of the growing number of cases. This delay reduced the fraction of cases that could be traced, resulting in a higher number of sporadic cases. After targeted group testing was implemented, the proportion with confirmation delay decreased, and hence more cases could be traced earlier. Together, with the tightening of social distancing measures, this increase in contact tracing efficiency contributed to suppression of the outbreak.

## Discussion

If effective contact tracing is essential for the control of outbreaks of COVID-19,^1^, ^2^, ^24^, ^27^, ^28^ maintaining efficiency in the face of changing demand is surely critical. However, no previous report has documented changes in contact-tracing efficiency throughout a substantial outbreak that was suppressed by NPIs. An important conclusion from our study of Hong Kong’s third wave is that the initial explosion of cases over-burdened the system of tracing and testing, delaying confirmation of infection. In a vicious circle, this increased delay led to reduction in the number of cases that could be traced and hence amplification of growth of the outbreak, which further delayed confirmation. During outbreak expansion, the proportion of all cases without an epi-link and of contact-traced cases with confirmation delay both increased rapidly (Figures 1A, 3A), as did the average duration of confirmation delay (Figure 3B). This strongly implies that reduction in contact-tracing efficiency exacerbated delays in confirmation and hence played a part in driving the outbreak.

The probability of confirmation delay was strongly associated with the rise in daily case number throughout the outbreak. Defining this ‘efficiency-load relationship’ in contact tracing gave us the opportunity to estimate the impact of a specific intervention – targeted group testing. A recent modelling study suggested that weekly screening of health-care workers and other high-risk groups, irrespective of symptoms, could reduce their contribution to transmission.^5^ Here, we provide observational evidence that the provision of targeted testing in high-risk groups had a substantial and sustained impact on efficiency, enabling the tracing and testing system to tolerate a 2.5-fold increase in daily case number before the percentage with confirmation delay rose above its previous value (Figure 5). Repeated, comprehensive mass screening could identify more cases, including asymptomatic cases, but the cost and workload would be higher.

Hence, our results highlight the importance of the following actions to effectively control an outbreak:

**i)** **Monitoring confirmation delay**. A high proportion with confirmation delay indicates low efficiency of testing and tracing. The efficiency of testing and tracing system can be weakened during outbreak growth and the following intervention is recommended to improve it, hence avoiding severe confirmation delay.
**ii)** **Testing at-risk people**. Testing at-risk people (i.e. people who have a higher probability of contact with an infection source) regardless of their symptoms reduces the delay of confirmation. Successful at-risk group testing requires the assessment of risks among different people in advance. Risk groups can be defined based on contact rates (e.g. certain occupational groups have more contacts) or contact location (e.g. people who live in the same building or community as infection sources).
**iii)** **Evaluating performance**. Severe confirmation delay occurs when the testing and tracing system reaches its capacity threshold (measured in case number). A higher capacity represents a better performance because the system can maintain its efficiency against a higher case number. For example, in Hong Kong, it appeared that when the outbreak was growing, the percentage with confirmation delay increased above 85%, indicating severe delay. Note that this critical percentage can be determined using a more stringent or relaxed criterion. After at-risk people were tested, the capacity threshold for avoiding severe delay improved from about 50 to 125 cases. Assessment of the capacity threshold is important for knowing whether an outbreak can be managed effectively without having such severe delay.

The proportion of transmission occurring prior to symptoms is an important factor to determine the potential for NPIs to stop the disease spread. If the level of presymptomatic transmission is high, conventional isolation and contact tracing approaches may still not control the spread easily.^29^ The occurrence of confirmation delay makes the task even harder. Therefore, putting appropriate resources on high-risk groups to early identify cases helps to control the outbreak. Implementing targeted testing can prevent these delays resulted from conventional contact tracing and testing. How to identify which groups having a high risk becomes a critical step.

We have evaluated the effects of some possible confounders in Hong Kong. Potentially, daily number of test conducted is likely to be a confounding factor. However, model comparison shows that after incorporating this number, effects of case number and targeted testing are still very similar. It is likely because in Hong Kong, number of tests conducted each day quickly reached their capacity. If the number is restricted, then the conditions for confounding to occur is not present. Furthermore, risk perception or being worried is likely not related to confirmation delay. Possibly people in Hong Kong were already very cautious since the early spread of COVID-19.^30^ It appears that the delay was mainly due to overload in testing and tracing.

The average duration of confirmation delay mirrored the changes in occurrence of delay until the end of the plateau, after which the average duration of delay increased, while the proportion without delay (‘negative delay’) continued to decrease (Figure 3A,B). This apparent paradox is clearly due to the appearance of a population of cases with longer delays (>4 days). At that stage, the public testing service was not able to deal with the demand created by targeted testing, and private service providers were engaged to help.^11^ Perhaps resources for timely testing were concentrated on contract-traced individuals who had not yet developed symptoms, while the delay for others increased because of the workload.

Two messages emerge. First, in addition to the fraction without an epi-link and the proportion with confirmation delay, average duration of delay is another useful indicator of efficiency, as long as the strategy for testing is consistent. Second, this provides evidence for the paramount importance of preparedness, with plans for the rapid mobilization and allocation of additional resources when needed. Maintaining sufficiency testing capacity is important. During this outbreak, the rate of total tests conducted (including targeted testing) is about 2.3 per thousand people (Figure S7), which are similar as the United Kingdom and the United States during summer,^31^ even though the case number was much lower than these countries.

Quantifying the risk (e.g. number of infections in a given period of time) of different types of transmission cluster can help to inform exit strategies.^32^ Our results imply that the risk associated with any cluster depends on not only the number of contacts made within the cluster, but also the ease and speed of tracing of close contacts.

Longer delays in confirmation should result in more secondary cases and higher transmission.^1^, ^2^, ^5^ This leads to an expectation that clusters with more delay should be larger, but a previous study^6^ found no obvious relationship between the confirmation delay and the size of clusters in the much smaller ‘second wave’ outbreak Hong Kong. Variation in timing, duration and shape of the clusters in the third wave (Figure 3A) made it difficult to infer a simple correlation between average confirmation delay and total case number across the lifetime of the clusters. In any case, severe delay is likely not only to increase transmission but also to reduce the probability of cases being traced. Many subsequent infections probably ’escaped’ from their cluster and became sporadic cases, eroding any correlation between delay and observed cluster size.

Without extensive case-investigation, contact tracing, and quarantine, some countries rely on other NPIs such as social-distancing (including city lockdowns, gathering ban, mask-wearing, etc) and precautionary measures (such as reducing face-to-face schooling, screening visitors at borders, etc) to control or prevent outbreaks. Observational data were sufficient to assess the impact of targeted testing on contact tracing, because of its substantial effect on preventing delays in confirmation. By comparison, epidemiological modelling is needed to estimate the impact of social distancing measures on infection rate and reproduction number. In the following paper^12^ we incorporate the changing efficiency of tracing and testing into a model, which allows the contributions of all the NPIs to be individually estimated.

One of the study limitations is that the lack of information on the exact cause of different delays, such as sample collection, waiting time for PCR testing, and time to trace close contacts. Knowing which factors contribute most to test and trace bottlenecks is important in order to reduce the delay. Whether there are issues of compliance with testing policies (e.g. people do not go to test even if they belong to high risk groups) is still unknown, which can be studied in the future. It is worth noting that from the data, we did not observe a minimum capacity, above which, delays began to worsen. One possible reason is that after the first index case was identified, the number of reported cases increased rapidly, overloading the capacity immediately. Therefore, delays only continued to worsen. In addition, how to determine an optimal allocation of testing resources between traditional contact tracing and targeted testing of at-risk people to increase the efficiency remains to be studied. Having more detailed data about the quarantine exempted persons can improve importation risk assessment in the future. Overall, an improved understanding about the relationship between confirmation delay and case number can provide a more accurate description of the infection dynamics.

### Data sharing

The source code and data for the work is available at https://github.com/hy39/tracing_hk_wave3.

### Author contributions

**Hsiang-Yu Yuan:** Data curation, Writing – original draft. **Colin Blakemore:** Conceptualization, Writing – original draft.

## Declaration of interests

All authors declare no competing interests.

## Appendix A Supplementary materials

Supplementary Data S1 Supplementary Raw Research Data. This is open data under the CC BY license http://creativecommons.org/licenses/by/4.0/ Supplementary Data S1

## References

[1] Hellewell J., Abbott S., Gimma A., Bosse N.I., Jarvis C.I., Russell T.W. (2020). Feasibility of controlling COVID-19 outbreaks by isolation of cases and contacts. Lancet Glob Health.

[2] Kretzschmar M.E., Rozhnova G., Bootsma M.C., van Boven M., van de Wijgert J.H., Bonten M.J. (2020). Impact of delays on effectiveness of contact tracing strategies for COVID-19: a modelling study. The Lancet Public Health.

[3] Tsou H.H., Cheng Y.C., Yuan H.Y., Hsu Y.T., Wu H.Y., Lee F.J. (2020). The effect of preventing subclinical transmission on the containment of COVID-19: Mathematical modeling and experience in Taiwan. Contemporary Clinical Trials.

[4] Gardner B.J., Kilpatrick A.M. (2020). medRxiv.

[5] Grassly N.C., Pons-Salort M., Parker E.P.K., White P.J., Ferguson N.M., Ainslie K. (2020). Comparison of molecular testing strategies for COVID-19 control: a mathematical modelling study. The Lancet Infectious Diseases.

[6] Adam D.C., Wu P., Wong J.Y., Lau E.H.Y., Tsang T.K., Cauchemez S. (2020). Clustering and superspreading potential of SARS-CoV-2 infections in Hong Kong. Nature Medicine.

[7] Kim H.J., Hwang H.S., Choi Y.H., Song H.Y., Park J.S., Yun C.Y. (2020). The delay in confirming COVID-19 Cases Linked to a Religious Group in Korea. Journal of Preventive Medicine and Public Health.

[8] To K.K.W., Chan W.M., Ip J.D., Chu A.W.H., Tam A.R., Liu R. (2020). Unique SARS-CoV-2 clusters causing a large COVID-19 outbreak in Hong Kong. Clinical Infectious Diseases.

[9] Siu G.K.H., Lee L.K., Leung K.S.S., Leung J.S.L., Ng T.T.L., Chan C.T.M. (2020). Will a new clade of SARS-CoV-2 imported into the community spark a fourth wave of the COVID-19 outbreak in Hong Kong?. Emerging Microbes and Infections.

[10] Chan W.M., Ip J.D., Chu A.W.H., Tse H., Tam A.R., Li X. (2021). Phylogenomic analysis of COVID-19 summer and winter outbreaks in Hong Kong: An observational study. The Lancet Regional Health - Western Pacific.

[11] Hong Kong Government. Government explains COVID-19 testing arrangements for target groups (6 August 2020). Available at https://www.info.gov.hk/gia/general/202008/06/P2020080600056.htm

[12] Yuan H.Y., Blakemore C. (2021). The impact of multiple non-pharmaceutical interventions on controlling COVID-19 outbreak without lockdown in Hong Kong: A modelling study. Lancet Regional Health Western Pacific.

[13] Hong Kong Centre for Health Protection. The latest situation of COVID-19 confirmed cases in HK (as of 25 October 2020). Available at https://www.chp.gov.hk/files/pdf/local_situation_covid19_en_20201025.pdf

[14] Department, Hong Kong Immigration. Statistics on Passenger Traffic. Available at https://www.immd.gov.hk/eng/message_from_us/stat_menu.html

[15] Li Q., Guan X., Wu P., Wang X., Zhou L., Tong Y. (2020). Early transmission dynamics in Wuhan, China, of novel coronavirus–infected pneumonia. New England Journal of Medicine.

[16] Government, Hong Kong. Government relaxes social distancing measures under Prevention and Control of Disease Ordinance (16 June 2020). Available at https://www.info.gov.hk/gia/general/202006/16/P2020061600761.htm

[17] Hong Kong Government. Government announces latest disease prevention measures (30 June 2020). Available at https://www.info.gov.hk/gia/general/202006/30/P2020063000854.htm

[18] Hong Kong Government. Government tightens social distancing measures (9 July 2020). Available at https://www.info.gov.hk/gia/general/202007/09/P2020070900723.htm

[19] Hong Kong Government. Government further tightens social distancing measures (14 July 2020). Available at https://www.info.gov.hk/gia/general/202007/14/P2020071400010.htm

[20] News.gov.hk. Mask rules enhanced (22 July 2020). Available at https://www.news.gov.hk/eng/2020/07/20200722/20200722_144255_095.html

[21] Government, Hong Kong. Government further tightens social distancing measures (27 July 2020). Available at https://www.info.gov.hk/gia/general/202007/27/P2020072700650.htm

[22] Weitz J.S., Park S.W., Eksin C., Dushoff J. (2020). Awareness-driven behavior changes can shift the shape of epidemics away from peaks and toward plateaus, shoulders, and oscillations. Proceedings of the National Academy of Sciences.

[23] Keeling M.J., Hollingsworth T.D., Read J.M. (2020). Efficacy of contact tracing for the containment of the 2019 novel coronavirus (COVID-19). Journal of Epidemiology and Community Health.

[24] Kucharski A.J., Klepac P., Conlan A.J.K., Kissler S.M., Tang M.L., Fry H. (2020). Effectiveness of isolation, testing, contact tracing, and physical distancing on reducing transmission of SARS-CoV-2 in different settings: a mathematical modelling study. The Lancet Infectious Diseases.

[25] Hong Kong Government. Progress of Targeted Group Testing Scheme (TGTS) (as of 17 September 2020). Available at https://gia.info.gov.hk/general/202009/19/P2020091900029_349682_1_1600449556925.pdf

[26] News.gov.hk. COVID-19 patient capacity boosted (29 July 2020). Available at https://www.news.gov.hk/eng/2020/07/20200729/20200729_181148_082.html

[27] Anderson R., Vegvari C., Maddren R., Baggaley R. (2020). SARS-CoV-2: Where do people acquire infection and ‘who infects whom’?. Royal Society.

[28] Rubin G.J., Smith L.E., Melendez-Torres G.J., Yardley L. (2020). Improving adherence to ‘test, trace and isolate’. Journal of the Royal Society of Medicine.

[29] Fraser C., Riley S., Anderson R.M., Ferguson N.M. (2004). Factors that make an infectious disease outbreak controllable. Proceedings of the National Academy of Sciences of the United States of America.

[30] Cowling B.J., Ali S.T., Ng T.W., Tsang T.K., Li J.C., Fong M.W. (2020). Impact assessment of non-pharmaceutical interventions against coronavirus disease 2019 and influenza in Hong Kong: an observational study. The Lancet Public Health.

[31] Ourworldindata.org. Coronavirus (COVID-19) Testing. Available at https://ourworldindata.org/coronavirus-testing;

[32] Leclerc Q.J., Fuller N.M., Knight L.E., Funk S., Knight G.M. (2020). What settings have been linked to SARS-CoV-2 transmission clusters?. Wellcome Open Research.

[33] Savvides C., Siegel R. (2020). medRxiv.

