# Supplementary material for: The impact of contact tracing and testing on controlling COVID-19 outbreak without lockdown in Hong Kong: An observational study
Source: Lancet Reg Health West Pac. 2022 Jan 15;20:100374. doi: 10.1016/j.lanwpc.2021.100374 (PMC8759949; doi:10.1016/j.lanwpc.2021.100374)
Supplement: Supplementary Data S1 — Supplementary Raw Research Data. This is open data under the CC BY license http://creativecommons.org/licenses/by/4.0/ [file mmc1.pdf]

Supplementary Material

The impact of contact tracing and testing on controlling COVID-19 outbreak without lockdown in Hong Kong: An observational study

Hsiang-Yu Yuan[1], Colin Blakemore[2,3]

1 Department of Biomedical Sciences
Jockey Club College of Veterinary Medicine and Life Sciences
City University of Hong Kong
Hong Kong SAR, China

2 Department of Neuroscience and
City University of Hong Kong
Hong Kong SAR, China

3 Hong Kong Institute for Advanced Study
City University of Hong Kong
Hong Kong SAR, China

Correspondence to
Hsiang-Yu Yuan: sean.yuan@cityu.edu.hk
or Colin Blakemore: colin.blakemore@cityu.edu.hk

Supplementary Material

Incorporating undetected imported cases

In order to calculate those undetected imported cases that were exempt from compulsory quarantine at entry, we assumed that the percentage of infected individuals was the same among visitors who were exempt from quarantine as among those who were quarantined. Because the exempt visitors were required to report to the Department of Health if any symptoms developed, 'undetected imported cases' refers to those exempt visitors who were infectious but asymptomatic, assuming that everyone complied with this requirement. The number of undetected imported cases in each day, t, can be calculated as:

$$\begin{aligned}\#Undetected\,imported(t) &= p_{asymp} \cdot \frac{\#Exempted(t)}{\#Visitors(t)} \cdot \#Total\,imported(t) \\ &= \rho(t) \cdot \#Total\,imported(t)\end{aligned} \tag{1}$$

where ρ is the product of the ratio of the number of exempt visitors ($\#Exempted$) to the total number of visitors ($\#Visitors$) and the percentage of asymptomatic cases p_{asymp}. We set p_{asymp} to be 17% according to a previous study [1]. Reported imported cases contain both non-exempt imported cases (symptomatic and asymptomatic cases) and exempt imported cases who developed symptoms. Total imported cases contain both reported imported cases and undetected imported cases, such that $\#Total\,imported = \#Reported\,imported(t) + \#Undetected\,imported(t)$. After rearrangement, we obtained:

$$\#Undetected\,imported(t) = \frac{\rho(t)}{1-\rho(t)} \cdot \#Reported\,imported(t) \tag{2}$$

The daily number of confirmed imported cases, $\#Reported\,imported(t)$, was collected from the Hong Kong Centre for Health Protection (CHP) [2]. Because exempt visitors were issued with medical surveillance notices by the Department of Health [3], the daily number of exempt visitors, $\#Exempted(t)$, was estimated using the medical surveillance notices issued at the border control points. From the daily number of visitors,

$\#Visitors(t)$, obtained from the Immigration Department, the proportion of the exempted visitors among total visitors was calculated as 56.1% in June. $\rho(t)$ was obtained by multiplying the proportion by p_{asymp}. A smoothed estimate of $\frac{\rho(t)}{1-\rho(t)}$ was 10.5% with an average daily increase rate of 0.6%, calculated using the data available from the Immigration Department [4].

Variable selection and model comparison

Besides the number of confirmed cases and targeted group testing, we investigated whether daily number of tests conducted, social distancing, face mask wearing, and risk perception (being worried) should be incorporated into our model (Figure S6). NPIs which affect the number of effective contacts (such as social distancing and face mask wearing), are independent of confirmation delay, and hence they cannot modify the relationship between the confirmation delay and case number. Therefore, these two factors were not included in our model. Instead, they were incorporated in our modelling study of NPIs [5].

We further investigated the impact of perceiving risk among cases in transmission clusters. Proportion test based on Chi-squared was performed to test whether more cases in transmission clusters were tested earlier as the clusters were formed. We divided clustered cases among two groups: early and late groups. If more people among transmission clusters were tested earlier because they were aware of the risk more, the percentage with confirmation delay were likely to become smaller. Among all clusters shown in Figure 2 in main text (483 cases in 15 clusters), we compared cases confirmed during the early period of each cluster (likely to have lower risk perception) with those confirmed during the late phase of each cluster (likely to have higher risk perception). There was no statistical difference between the cases identified earlier (83.5% with confirmation delay) and later (80.4% with confirmation delay) (p-value = 0.459 using two-sided tests). The time at which the close contacts of a case were traced and tested was mainly controlled by the Hong Kong government and the testing capacity. It appeared that confirmation delay was not caused by the change in risk perception.

Next we evaluated whether the daily number of tests conducted is likely to be a confounding factor. We developed an alternative model after incorporating the number of tests conducted each day (Figure S7):

$$log(\frac{\mu_t}{1-\mu_t}) = \alpha + \beta \times Cases_t + \beta_T \times Tests_t + \gamma \times (Cases_t \cdot D_t) \quad (3)$$

where $log(\frac{\mu_t}{1-\mu_t})$ is the logit of the probability of having confirmation delay μ at day t. β_T is the regression coefficient of the average daily number

of tests conducted (without targeted testing), $Tests_t$. Other variables are same as those in the original model (equations 1 and 2 in the main text).

Model comparison shows that after $Tests_t$ was included, parameter values (i.e. β and γ) were still very similar as the original model (Table S2). Less than 3% differences were produced in both predictors. Further more, model fitting using Quasi AICc, a modified version of corrected Akaike's Information Criterion for overdispersed count data shows that the original model with case numbers and targeted testing is the best-fitting model.

Supplementary Tables and Figures

Table S1: Mobility of the public during implementation of significant social distancing measures. See main text for a description of each intervention. Mobility describes the time spent in two categories of location: **Transit stations** and **Retail & recreation**. T0 refers to social distancing regulations introduced on June 5 and in place at the time of the first relaxation (R1). Daily mobility data [6] were normalized after setting the average mobility between T0 and R1 as the baseline (100%). The average daily mobility for T0, R1 and R2 was calculated over the period from each intervention until the next. The average daily mobility for T1, T2 and T4 was calculated over the first 3 days following the introduction of each intervention.

**Intervention**	**Number of people permitted in public places**	**Number permitted in catering premises and places of entertainment**	**Mobility (Transit stations; %)**	**Mobility (Retail & recreation; %)**
**T0**	8	8	100	100
**R1**	50	50	103	105
**R2***	50	50	104	105
**T1**	50	8	95	96
**T2**	4	4	90	83
**T4**	2	2	77	63

* There was no change in gathering limits for R2, but the maximum number of people in places of entertainment was raised from 50% to 80% of capacity (see main text).

Table S2: The QAICc of each model with the selected predictors. The mean value of each predictor is shown with standard deviation (SD). QAICc was calculated using regular model with the extracted overdispersion parameter (see [7]).

Model	Formula	Predictor values	QAICc
Original	$\alpha + \beta Cases_t + \gamma(Cases_t \cdot D_t)$	β = 0.029806 (SD=0.009234); γ = -0.017877 (SD=0.007308)	133.11
Alternative	$\alpha + \beta Cases_t + \beta_T Tests_t$ $+ \gamma(Cases_t \cdot D_t)$	β = 0.02911 (SD=0.009371); β_T = 0.0000267 (SD=0.00004219); γ = -0.01782 (SD=0.007376)	134.14

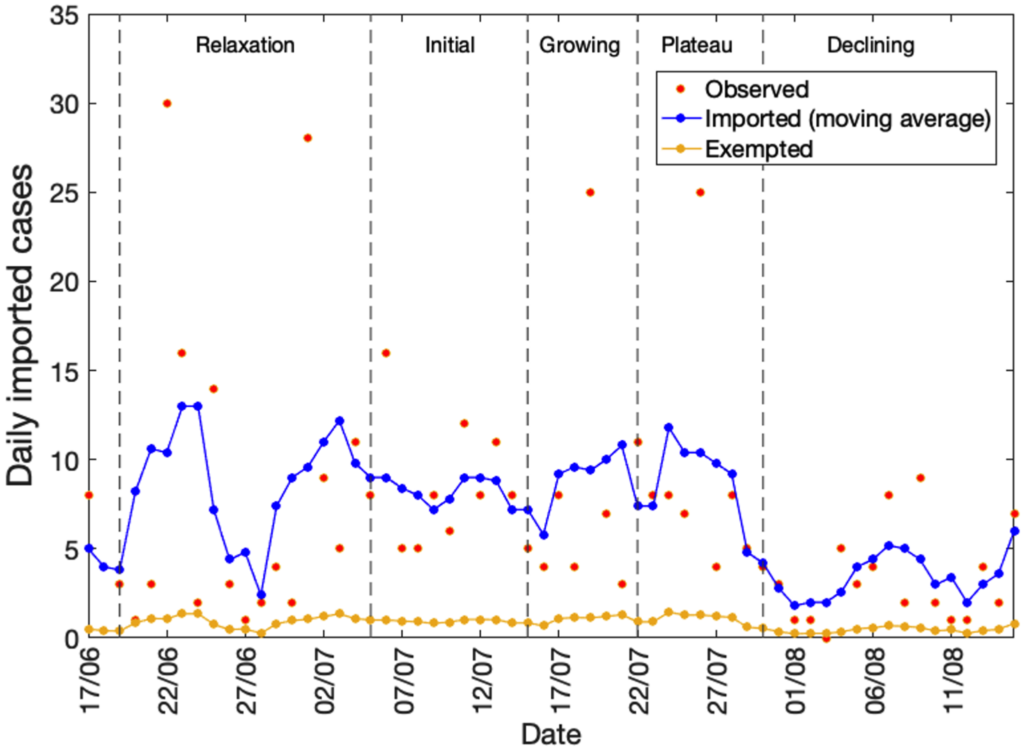

Figure S1 Number of daily reported imported cases. The red circles represent the number of actual daily reported imported cases. The blue line is the moving average. The brown circles and line represents the expected daily number of imported cases that were exempted (and undetected).

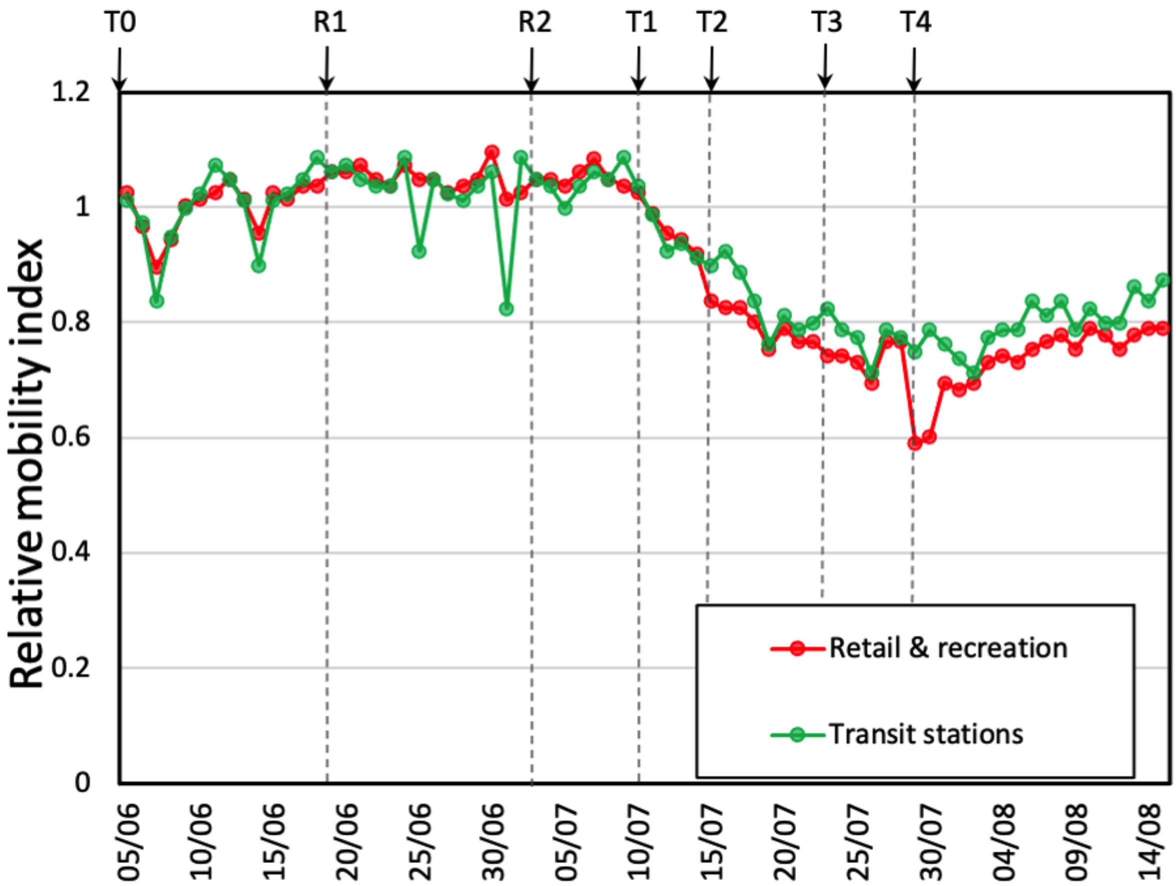

Figure S2 Relative mobility index during the outbreak. Daily mobility data from COVID-19 Community Mobility Reports [6] were normalized after setting the average mobility between T0 and R1 to a baseline value of 1. Red and green circles and lines represent, respectively, the mobility index in **Retail & recreation** and in **Transit stations**.

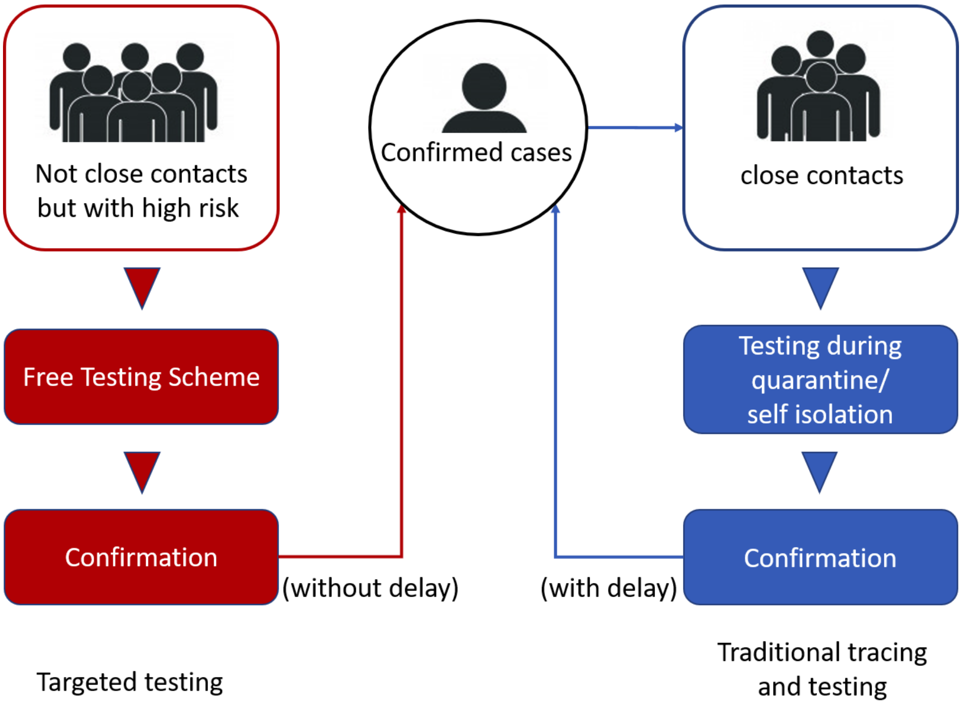

Figure S3 Traditional tracing and testing and targeted testing. Tests are conducted for close contacts in the traditional approach (blue). Individuals who are not close contacts but belong to high risk groups are tested before symptom onset (red).

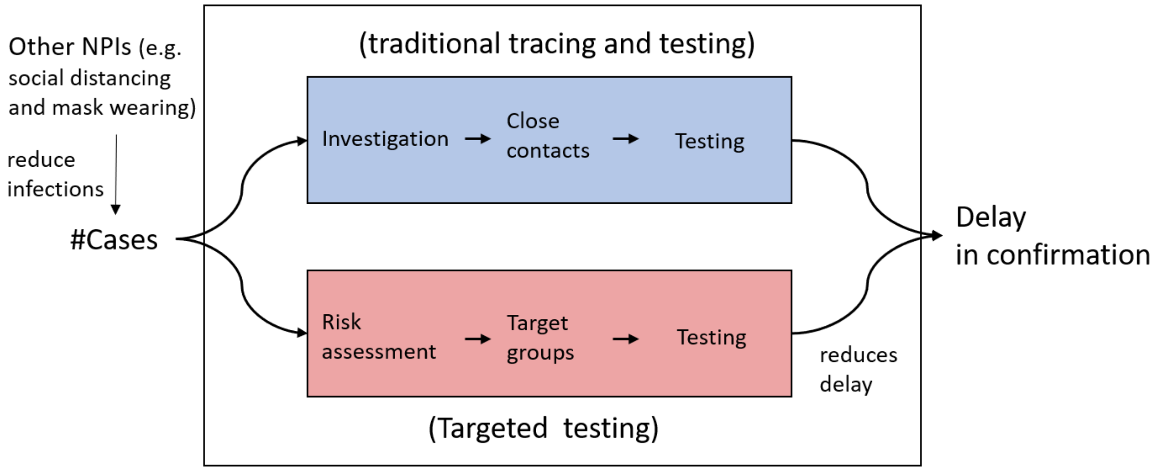

Figure S4 Schema of the relationship between number of cases and confirmation delay in traditional testing and targeted testing approaches.

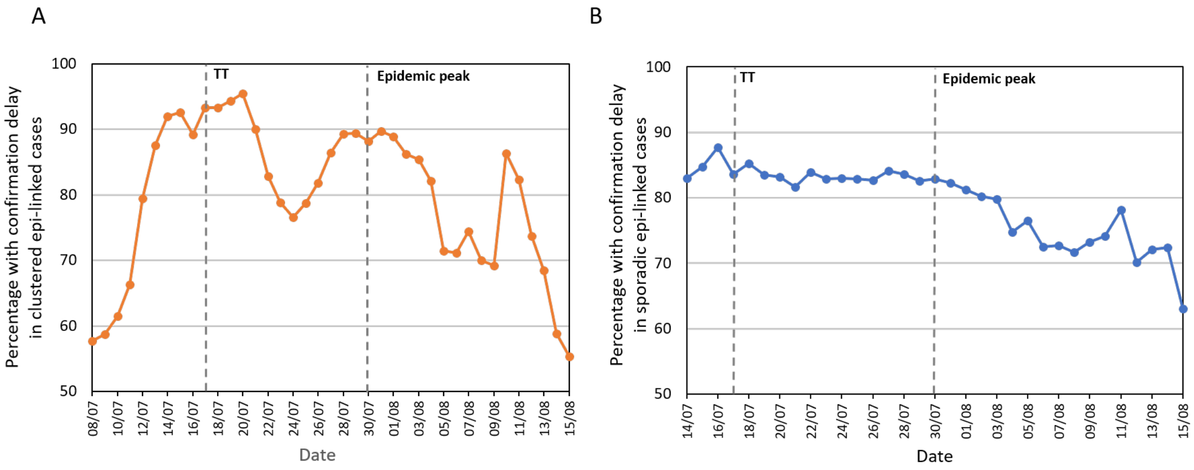

Figure S5 Percentages with confirmation delay in epi-linked cases in clusters (A) and epi-linked cases not in clusters, also called sporadic epi-linked cases (B). 5-day average of percentage with confirmation delay is plotted after the average daily number of confirmed cases is greater than 10.

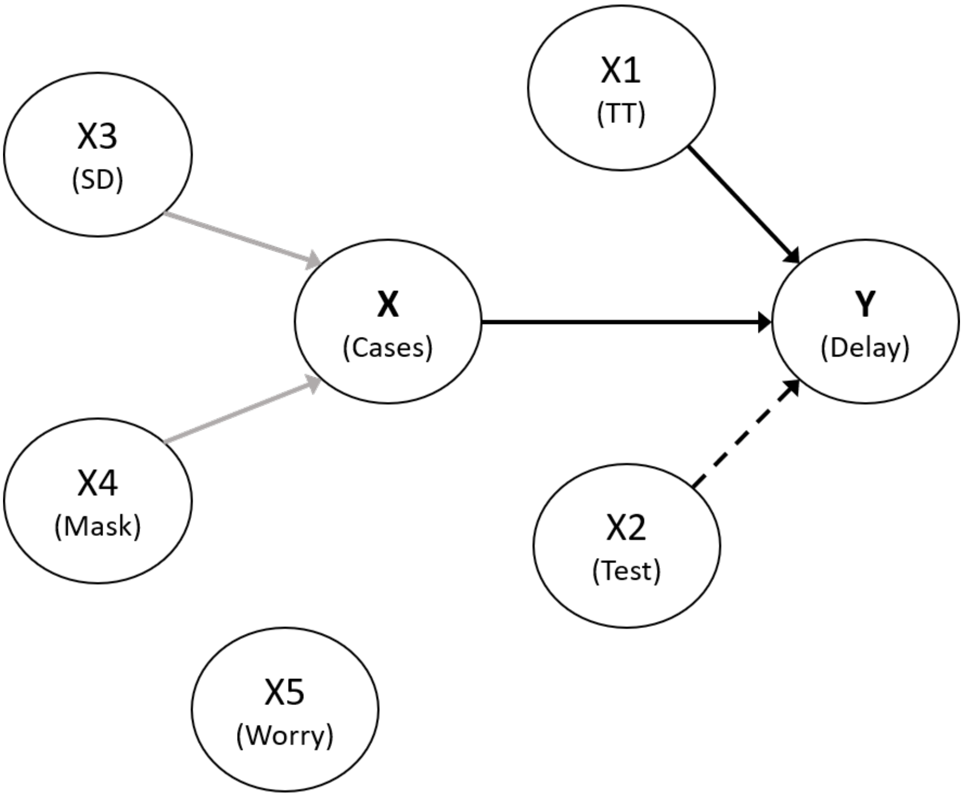

Figure S6 Diagram of predictors and other factors for producing confirmation delay. Solid black lines indicate the established relationship in the best-fitting model. Gray lines refer to connections that do not affect the model. Dashed line refers to connections that do not have significant effect. (Cases = number of confirmed cases; TT = targeted testing; Test = daily number of tests (not including those from targeted testing); SD = social distancing measures; Mask = face mask wearing regulation; Delay = confirmation delay).

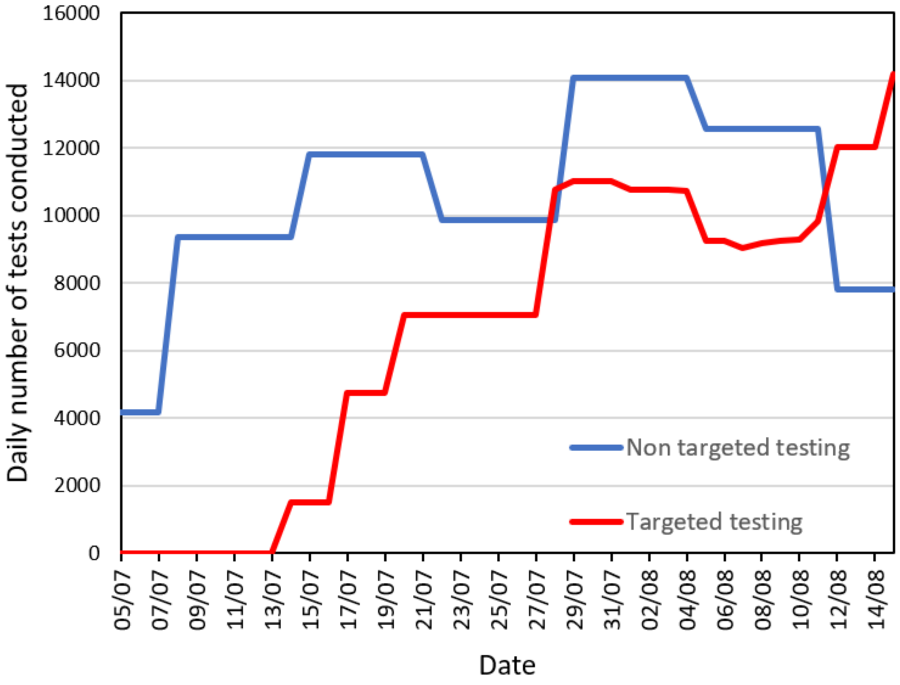

Figure S7 Statistics on testing for COVID-19. Blue indicates the number of tests conducted without targeted testing. Daily number in blue curve was calculated from weekly statistics [8]. Red indicates the number in targeted testing. Daily number in red curve was calculated from the average daily number of tests in each of the phases of targeted testing [9]. Average daily number of total tests conducted during this period is 17258, corresponding to 2.3 per one thousand people assuming the total population is 7.5 million.

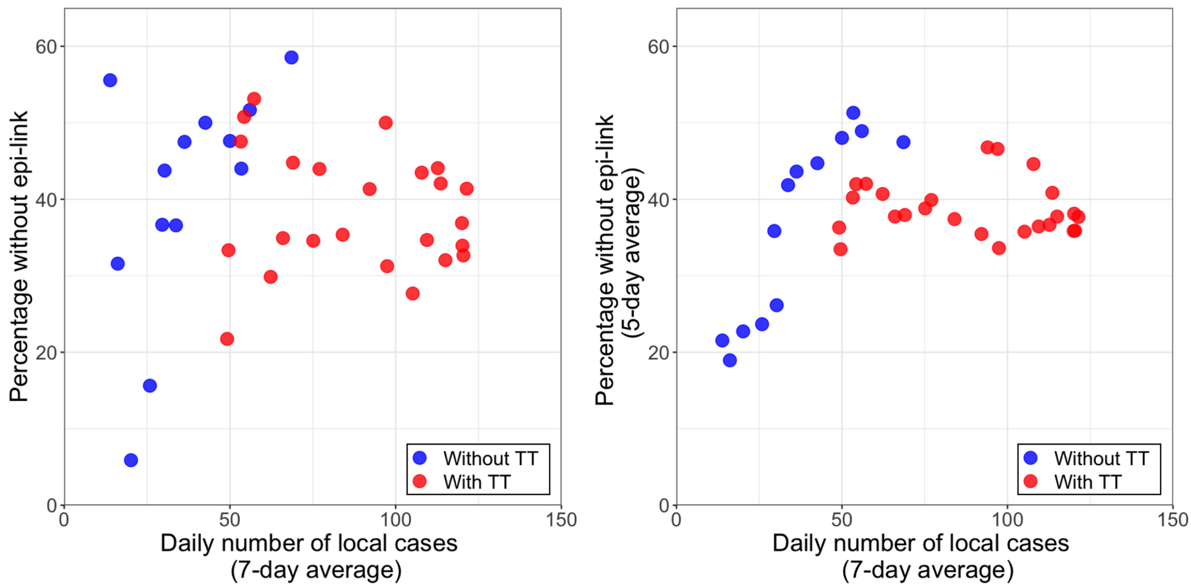

Figure S8 Percentage of cases without an epi-link as a function of the daily number of cases (average by week), displayed as for the analysis of confirmation delay in Figure 5. (A) Data plotted on linear coordinates. Blue and red circles describe data before and after the introduction of targeted group testing (TT, see main text). (B) To reduce the impact of daily variation in the number of reported cases, the 5-day average of percentage without an epi-link is plotted on the ordinate. The trend shown in (B) is similar to that for the occurrence of confirmation delay (see *Impacts of targeted group testing* in main text): the percentage without an epi-link is consistently lower after TT, across the range of case number, and the slope of the relationship appears steeper before TT than after. However, variability is high and the predicted fits from logistic regression did not reach statistical significance.

References

[1] Oyungerel Byambasuren, Magnolia Cardona, Katy Bell, Justin Clark, Mary-Louise McLaws, and Paul Glasziou. Estimating the extent of asymptomatic COVID-19 and its potential for community transmission: Systematic review and meta-analysis. *Official Journal of the Association of Medical Microbiology and Infectious Disease Canada*, 5(4):223–234, 2020.

[2] Hong Kong Centre for Health Protection. The latest situation of COVID-19 confirmed cases in HK (as of 29 April 2020). Available at https://www.chp.gov.hk/files/pdf/local_situation_covid19_en.pdf.

[3] Hong Kong Government. Government supplements on quarantine exemption arrangement (26 July 2020). Available at https://www.info.gov.hk/gia/general/202007/26/P2020072600666.htm.

[4] Hong Kong Immigration Department. Statistics on Passenger Traffic. Available at https://www.immd.gov.hk/eng/message_from_us/stat_menu.html.

[5] Hsiang-Yu Yuan and Colin Blakemore. The impact of multiple non-pharmaceutical interventions on controlling COVID-19 outbreak without lockdown in Hong Kong: A modelling study. *The Lancet Regional Health – Western Pacific*, 0:100343, 2021.

[6] Google LLC. COVID-19 Community Mobility Reports , 2020. Available at https://www.google.com/covid19/mobility, 2020.

[7] Ben Bolker. Dealing with quasi-models in R. Available at https://cran.r-project.org/web/packages/bbmle/vignettes/quasi.pdf, 2021.

[8] DATA.GOV.HK. Data in Coronavirus Disease (COVID-19). Available at https://data.gov.hk/en-data/dataset/hk-dh-chpsebcddr-novel-infectious-agent, 2020.

[9] Hong Kong Government. Government explains COVID-19 testing arrangements for target groups (6 August 2020). Available at https://www.info.gov.hk/gia/general/202008/06/P2020080600056.htm.
